# Down to the bone: the role of overlooked endolithic microbiomes in reef coral health

**DOI:** 10.1038/s41396-019-0548-z

**Published:** 2019-11-05

**Authors:** Mathieu Pernice, Jean-Baptiste Raina, Nils Rädecker, Anny Cárdenas, Claudia Pogoreutz, Christian R. Voolstra

**Affiliations:** 1 Climate Change Cluster, University of Technology Sydney, Sydney, NSW Australia; 2 Red Sea Research Center, Biological and Environmental Sciences and Engineering Division (BESE), King Abdullah University of Science and Technology (KAUST), Thuwal, Saudi Arabia; 3 Department of Biology, University of Konstanz, 78457 Konstanz, Germany

**Keywords:** Water microbiology, Microbial ecology, Symbiosis

## Abstract

Reef-building corals harbour an astonishing diversity of microorganisms, including endosymbiotic microalgae, bacteria, archaea, and fungi. The metabolic interactions within this symbiotic consortium are fundamental to the ecological success of corals and the unique productivity of coral reef ecosystems. Over the last two decades, scientific efforts have been primarily channelled into dissecting the symbioses occurring in coral tissues. Although easily accessible, this compartment is only 2–3 mm thick, whereas the underlying calcium carbonate skeleton occupies the vast internal volume of corals. Far from being devoid of life, the skeleton harbours a wide array of algae, endolithic fungi, heterotrophic bacteria, and other boring eukaryotes, often forming distinct bands visible to the bare eye. Some of the critical functions of these endolithic microorganisms in coral health, such as nutrient cycling and metabolite transfer, which could enable the survival of corals during thermal stress, have long been demonstrated. In addition, some of these microorganisms can dissolve calcium carbonate, weakening the coral skeleton and therefore may play a major role in reef erosion. Yet, experimental data are wanting due to methodological limitations. Recent technological and conceptual advances now allow us to tease apart the complex physical, ecological, and chemical interactions at the heart of coral endolithic microbial communities. These new capabilities have resulted in an excellent body of research and provide an exciting outlook to further address the functional microbial ecology of the “overlooked” coral skeleton.

## Looking below the surface: abundance and diversity of endolithic communities

Endolithic environments—the pore spaces within solid substrates—are ubiquitous habitats for microbial life on Earth [1]. In terrestrial systems, these microenvironments typically provide protection from intense solar radiation and desiccation, as well as sources of nutrients, moisture, and substrates derived from minerals [2, 3]. In marine systems, endolithic communities similarly exploit the rocky seafloors, but also dwell into limestone and mineralised skeleton of a broad range of marine animals [4, 5]. A wide spectrum of boring microorganisms was already described in the late 1880s, with several species of cyanobacteria, fungi, and eukaryotic green algae known to penetrate coastal carbonate rocks and the shells of molluscs [6]. Coral endolithic microorganisms forming distinct and visible bands in the skeleton were first characterized not long after, in 1902 [7], a mere 19 years after the description of the unicellular symbiotic algae in coral tissues [8]. While the past 120 years have seen a vast improvement in our understanding of the ecology, physiology, metabolism, diversity, and genetics of Symbiodiniaceae [9], the photosynthetic microalgal symbionts inhabiting the tissue of corals, less effort was channelled into the characterization of endolithic microorganisms. Their ecological significance remains underexplored and detailed descriptions of their in situ microenvironment and activity are still scarce.

### Endolithic microalgae

The dense green band in the skeleton that can be observed underneath the tissue of many coral species is often dominated by the filamentous green algae *Ostreobium* spp. (Siphonales, Chlorophyta) [10]. This diverse genus can penetrate both dead carbonate substrates as well as live corals [11], and has been recorded in the aragonite skeletons of Atlantic, south Pacific, and Caribbean reef corals including *Pocillopora* spp., *Stylophora* spp., *Acropora* spp., *Favia* spp., *Montastrea* spp., *Porites* spp., and *Goniastrea* spp. [11–18]. Recent molecular studies have revealed the astonishing genetic diversity of this group, with up to 80 taxonomic units at the near-species level [19–22]. High-throughput amplicon sequencing has also revealed the presence of other, less abundant, boring green microalgae closely related to *Phaeophila*, *Bryopsis*, *Chlorodesmis*, *Cladophora*, *Pseudulvella*, and red algae from the Bangiales order in coral skeletons [19].

### Endolithic fungi

Endolithic fungi are as prevalent as microalgae in coral skeletons. They penetrate the calcium carbonate microstructures and ultimately interact with *Ostreobium* cells [23]. The first endolithic fungi isolated from coral skeletons in the Caribbean and the South Pacific belonged to the divisions Ascomycota and Basidiomycota [24]. The intrusion of fungal filaments into the polyp zone of the hermatypic coral *Porites lobata* is known to prompt a defence mechanism involving a dense deposition of skeleton, resulting in pearl-like structures [23]. These observations were extended to acroporid and pocilloporid corals, suggesting that endolithic fungi are geographically and taxonomically widespread [25]. Along with endolithic algae, fungi are present in the newly deposited coral skeleton [5, 25], and exhibit rapid growth to match skeletal accretion [23].

### Endolithic prokaryotes

Only a few studies have characterized the diversity of coral endolithic bacteria, but often ignored the potential spatial heterogeneity within this coral compartment. Species of filamentous marine cyanobacteria, such as *Plectonema terebrans*, *Mastigocoleus testarum*, and *Halomicronema excentricum*, were some of the first described prokaryotes from green bands of coral skeletons. These cyanobacteria were initially observed in shells of mussels and barnacles and subsequently found in association with a great diversity of corals [11, 26–28]. High abundance of cyanobacteria and anoxygenic phototrophic bacteria was suggested by spectral signatures of bacteriochlorophylls in the green and deeper bands of coral skeletons [18, 29]. Diversity surveys based on 16S rRNA gene amplicon sequencing have revealed more than 90 unclassified cyanobacterial OTUs (>97% similarity cut off) across the skeleton of 132 coral fragments [19]. In addition, anaerobic green sulphur bacteria from the genus *Prosthecochloris* were found prevalent in the skeleton of *Isopora palifera* [30].

## Physicochemical characteristics of the coral skeleton

The coral skeleton is a porous substrate with unique physicochemical characteristics [29, 31–33], which are derived from the influence of the overlying coral tissue and the internal structure of the skeleton itself. These specific physicochemical characteristics likely drive the spatial structure, interspecies interactions, ecophysiology, and functions of coral endolithic communities.

### Light

Photosynthetically active radiation (PAR)—the waveband of solar radiation ranging from 400 to 700 nm used by most algae for photosynthesis—are strongly attenuated by absorption from the Symbiodiniaceae cells within the coral tissue and intense scattering from the skeleton [34]. Earlier estimates suggested that up to 99% of the incident PAR were absorbed or scattered before reaching the endoliths [14, 35, 36]; similar values were derived from more recent in situ measurements, with 0.1–10% of incident PAR reaching the endolithic communities [29]. Besides water depth, internal irradiance is also strongly influenced by coral species through variation in tissue thickness and skeletal morphology [29].

Although endolithic oxygenic phototrophs subsist in extremely low levels of PAR, their light environment in shallow waters is not as depleted in near-infrared radiation (NIR) (wavelengths from >700 to 1000 nm) [29], as NIR is not absorbed by the coral tissue and penetrates much deeper into the skeleton [18]. These wavelengths can be exploited by bacteria harbouring bacteriochlorophyll which sustains anoxygenic photosynthesis [37], and by other long wavelength-absorbing pigments (e.g., chlorophyll d and f) in oxygenic phototrophs [38, 39]. However, since irradiance attenuation increases for higher wavelengths, the amount of NIR available for the endolithic communities is strongly constrained by water depth. Therefore, NIR can only sustain photosynthetic activity in reefs shallower than 15 metres [29].

### Oxygen and pH

Photosynthesis and respiration from endolithic communities generate diurnal fluctuations in the pH and oxygen concentrations within the skeleton [32, 33]. Due to the lower metabolic rate of endolithic microbes, these fluctuations are not as extreme as the ones recorded in coral tissues (which can fluctuate diurnally from pH 6.6 to 8.5 [32], and from nearly anoxic to 400% air saturation [33]). At night, heterotrophic respiration lowers oxygen levels (10–60% of ambient concentration) and pH (~7.69) in the pore water of the skeleton [31, 40], while during the day, photosynthetic activity triggers an increase of both oxygen (>210% of ambient concentration) and pH (over 8.5) [41]. However, the distribution of oxygen within the endolithic environment is not homogenous. Oxygen production predominantly occurs within the green *Ostreobium*-dominated band, while consumption, although greater in the zone directly below the corallites, does not exhibit a well-defined permanent zonation in the skeleton, indicating large heterogeneity in endolithic respiration [15]. In particular, the skeleton of the hydrocoral *Millepora*, which is highly porous but has a relatively low permeability, can trap large amounts of oxygen produced by endoliths. This oxygen can often be seen bubbling out of broken branches [42].

### Chemical gradients

Pore water within the skeleton differs drastically from the surrounding seawater in its chemical profile. High enrichment in phosphate, ammonium, nitrate, and nitrite have been recorded [31, 40, 43]. Although the metabolic activity of the overlying coral tissue undoubtedly will have an influence on the chemical composition of skeletal pore water, the diverse endolithic assemblages are likely responsible for the remineralization of organic matter and excretion of nutrients [31]. This enriched pore water has been, until now, completely overlooked and could constitute a potential source of nutrients for the tissue, but also for the Symbiodiniaceae. For instance, dissolved inorganic nitrogen concentrations present in pore water could fulfil 200% of the coral’s nitrogen demand [31]. It is however currently unknown if this enormous nutrient pool is accessible to the coral tissue.

### Species-specific features

The structure, density, and pore sizes of the skeleton can vary widely within and between coral species [44–46]. These differences in physical characteristics of the skeleton directly impact the light, oxygen, pH, and chemical microenvironments and therefore likely influence the distribution and structure of the endolithic communities [47]. For example, dense skeletons cause higher light attenuation compared to skeletons that are less dense. Consequently, denser skeletons more strongly promote the development of anoxic microenvironments, favouring anaerobic microorganisms [47]. Further, skeletal density is dependent on the growth form of the coral colonies: the densest skeletons can be found in foliaceous (i.e., thin and leaf-like) forms of the genera *Agaricia*, *Leptoseris*, *Orbicella*, or *Dichocoenia*. The most porous skeletons are typically associated with massive and bushy growth forms such as *Acropora* or *Seriatopora* [48]. It is also important to note that some branching species exhibit strong axial gradients in density [48], resulting in variations in skeletal porosity from 40 to 70% within the same *Acropora* colony skeleton [44].

**Fig. 1:**
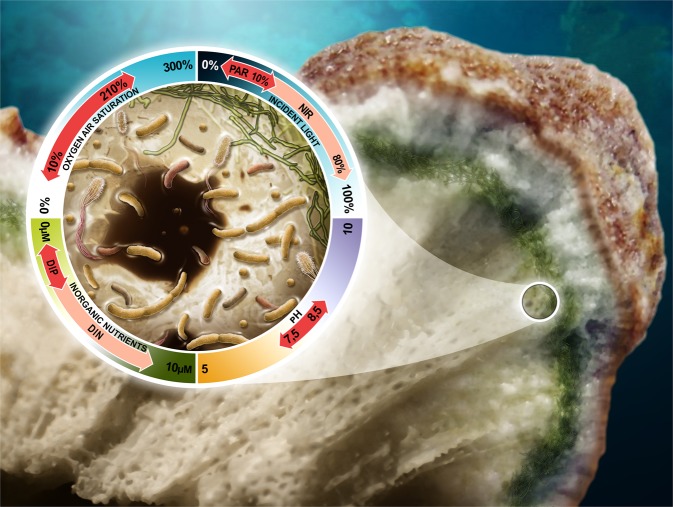
Spatial structure and physicochemical environment experienced by microbes within the coral skeleton. The close-up depicts a typical skeletal pore populated by a range of autotrophic (green) and heterotrophic microbes (other colours). These organisms typically experience daily fluctuations in pH (from 7.5–8.5) [41], oxygen (10–210% air saturation) [15] and light (0–10% of PAR and 0–80% of NIR) [29]. In addition, they are exposed to enriched levels of dissolved inorganic phosphorus (DIP) and to concentrations of dissolved inorganic nitrogen (DIN) 10 times higher than in reef water [31, 40]. Outline of the coral from [54]

Taken together, the skeletal environment is characterised by its extremely low ambient light, daily fluctuation in pH and oxygen, and enrichment in inorganic nutrients that are typically limiting primary production in reef water (Fig. 1). The coral skeleton is a porous structure and species-specific differences in pore size, shape, and volume can result in ~25–50% of “empty” space filled by water [49]. These pores divide the skeleton into numerous microhabitats in which slightly different microbial assemblages and metabolic processes might occur. It is therefore very likely that microscale heterogeneity in chemical, light, or pH might stratify the endolithic microbiomes, ultimately promoting spatially diverse and dynamic microorganism assemblages [21]. However, this heterogeneity might have been masked by the relatively low spatial resolution of most of the analyses conducted until now.

## Contribution and function of the endolithic microbiome to reef coral health

Recent molecular studies using amplicon sequencing of multiple marker genes (e.g., 16S rDNA, 18S rDNA, 23S rDNA, *tuf*A) have provided many new insights into the diversity of microbes associated with the coral skeleton [19–21, 30, 50]. However, in situ functional characterization of endolithic microbes has so far only been inferred via the amplification of targeted functional genes [30]. Earlier studies suggested that the complex assemblages of microorganisms populating the skeletons may influence coral health and disease mainly via their important role in bioerosion, primary production, and nutrient cycling.

### Primary productivity

Coral reefs are among the most productive marine ecosystems on Earth and several lines of evidence indicate that endolithic communities might contribute significantly to the high primary productivity of coral reefs. For example, the role of endolithic *Ostreobium* as primary producers was first demonstrated using incubation with ^14^C-bicarbonate [51]. Indeed, these algae assimilate inorganic carbon during the day and transfer photoassimilates (as lipids) into the tissues of the azooxanthellate coral *Tubastrea micranthus* within 24–48 h. Using similar methodology, Fine and Loya demonstrated the transfer of photoassimilates from endoliths to the coral tissues in the Mediterranean coral *Oculina patagonica* [52] and in zooxanthellate corals from the Great Barrier Reef [53], suggesting high primary production by these endolithic communities. Several studies have since supported the role of endolithic communities as primary producers in *Porites* corals [54], exceeding 40% of holobiont productivity in some instance [55]. In contrast, a number of studies have proposed that primary productivity and transfer of carbon by endoliths to coral hosts is rather limited [41, 56]. For instance, oxygen production measurements of endoliths in coral colonies suggested that the endolithic contribution accounted for <4% of the total primary production of corals [41]. Further, Titlyanov et al. also proposed that transfer of photoassimilates from endoliths to the host tissues of *Porites lutea* and *P. cylindrica* was limited to sunlit shallow waters [56]. While it is becoming increasingly clear that endolithic communities potentially play a significant role in coral reef primary productivity, it is important to note that this role may vary greatly with environmental conditions [57]. Further work is therefore needed to better understand the contribution of endolithic microbes to the overall carbon budget and primary productivity in corals.

### Nitrogen cycling

Given that most coral reefs inhabit waters where planktonic food supplies and dissolved nitrogen can be limiting, the ability to assimilate nitrogen and cycle it rapidly is crucial for the coral holobiont [58]. The first evidence for nutrient cycling within the coral skeleton dates back to 1955 [59]. Based on measurements of endolithic biomass and chlorophyll a concentration, it was hypothesized that the products of coral host metabolism and excretion could diffuse through the porous skeleton to benefit the endoliths, while the coral host could benefit in return from diffusion of organic substances produced by the endoliths [59]. However, the coral tissue and associated Symbiodiniaceae are a net sink for inorganic nitrogen, which might limit the diffusion of these compounds from the tissue into the underlying skeleton [60]. Consequently, the high concentrations of inorganic nitrogen within the pore water of coral skeletons suggests that endolithic microbes actively accumulate and efficiently recycle the nitrogen within this environment. It has been estimated that endoliths may satisfy 55–65% of nitrogen required by corals for balanced growth [31]. Indeed, coral endolithic microbial communities include a diversity of prokaryotic and eukaryotic microbes capable of driving key steps of the nitrogen cycle including nitrogen fixation and nitrification.

Nitrogen fixation refers to the conversion of dinitrogen (N_2_) molecules into ammonia (NH_3_) and occurs through a complex reductive process involving the activity of the nitrogenase enzyme. Using acetylene reduction measurements, Shashar et al. were the first to show that the microbes associated with the skeleton of massive faviid corals can fix N_2_ and act as a significant source of nitrogen for the coral host [61]. These results were supported by a more recent quantification of N_2_ uptake by endolithic communities [54, 62]. Cyanobacteria were long thought to be the key players mediating nitrogen fixation in endolithic microbial communities, because of their abundance in coral skeletons and the previous report of nitrogen fixation by unicellular cyanobacteria within the tissue of *Montastraea cavernosa* [63]. However, recent studies have also identified the presence of ubiquitous populations of green sulfur bacteria, capable of anoxygenic photosynthesis and nitrogen fixation, in the coral skeleton [30, 64]. These green sulfur bacteria harbour genes involved in nitrogen metabolism, including glutamine/glutamate synthases, reduction of hydroxylamine, and nitrogen fixation [47]. The ability of these bacteria to fix N_2_ was indeed confirmed in cultures using acetylene reduction assays and nanoscale secondary ion mass spectrometry (NanoSIMS) [47].

While nitrogen fixation is an important source of ‘new’ nitrogen within the coral holobiont [65], this strictly prokaryotic process is highly energy consuming. Hence, efficient recycling and retention of fixed nitrogen are critical to fulfill the nitrogen requirements of endolithic microbes and potentially the overlying coral tissue. Nitrification, the aerobic oxidation of ammonium into nitrite and nitrate, is an important component of nitrogen cycling in corals and may regulate nitrogen availability for Symbiodiniaceae [66]. Nitrate concentrations may exceed ammonium concentrations by more than-tenfold in skeletal pore water [40], suggesting that nitrifying microbes may play a critical role in controlling nitrogen availability within the coral skeleton. Heterotrophic fungi, ubiquitous members of the coral skeleton, are able to reduce nitrate and nitrite into ammonium and assimilate the latter for biosynthesis [67]. Together, this interplay of prokaryotic and eukaryotic nitrogen cycling processes may efficiently accumulate and conserve nitrogen within the coral skeleton. Indeed, the interaction of anaerobic (e.g., nitrogen fixation) and aerobic (e.g., nitrification) processes may be supported by the high spatial and temporal variability in oxygen concentrations within the coral skeleton [15]. Endolithic microbes may thus be able to fulfill their nitrogen requirements despite the limited supply of inorganic nitrogen from the surrounding seawater and coral tissue.

### Bioerosion

Bioerosion, the biogenic dissolution of CaCO_3_ [68], is one of the main destructive forces of coral reef structures and is driven by a diversity of macro- and micro-organisms, including bacteria, cyanobacteria, algae, and fungi [69, 70]. The main microboring taxon, *Ostreobium*, can dissolve up to 0.9 kg of CaCO_3_ per m^2^ of reef per year [71].

Our understanding of microbioerosion is still very incomplete. The acidic nature of metabolic by-products released by bioeroding microbes was long thought to be responsible for this process [72]. However, most members of the endolithic microbial community are photosynthetic (Chlorophyta, Rhodophyta, and Cyanobacteria), which typically increases the pH and precipitates carbonates instead of dissolving them [73]. Alternative mechanisms to explain the paradox of boring phototrophic microbes have more recently been proposed [74, 75]. A series of seminal papers used imaging and molecular techniques to demonstrate that the activity of the bioeroding cyanobacterium *Mastigocoleus testarum* was based on shifting the dissolution equilibrium by lowering Ca^2+^ concentration [75–78]. Indeed, this cyanobacterium uses a combination of transporters to take up Ca^2+^ at the excavation front, promoting dissolution of CaCO_3_ locally, and to further transport and excrete Ca^2+^ away. It remains unclear though if this mechanism is common in other endolithic cyanobacteria and microboring algae [78].

## Do endolithic microbes affect coral survival after stressful events?

Corals are susceptible to a range of stressors, including the effects of global climate change [79, 80]. Coral bleaching is a general stress response of corals, but in recent decades has been most commonly observed during prolonged high-temperature anomalies, such as particularly severe El Niño/Southern Oscillation events [81, 82]. The term bleaching refers to the disruption of the coral-algae symbiosis caused by the loss of photopigments or endosymbiotic dinoflagellates from the animal tissues [79, 83]. Bleaching thereby rapidly deprives the coral host of its main energy source, specifically photoassimilates translocated by the endosymbiotic dinoflagellates [84–87]. When symbiotic corals undergo bleaching, endolithic microbial communities, in particular, *Ostreobium* spp. exhibit pronounced responses, which have been attributed to the stark increase in PAR, as increasing light is able to penetrate the translucent coral tissues [53]. Exposed to low levels of PAR under normal conditions [14, 36, 51], the endolithic communities can rapidly photoacclimate during bleaching [53, 88] and subsequently ‘bloom’ due to increased light availability [89]. As a result, biomass, photosynthetic pigments, and rates of photoassimilate translocation can increase in the skeleton of bleached corals compared to non-bleached corals [52, 90]. This observation suggests that endolithic phototrophic microbes may indeed constitute a key supply of energy for the stressed coral animal, potentially supporting survival or even recovery following bleaching.

**Fig. 2:**
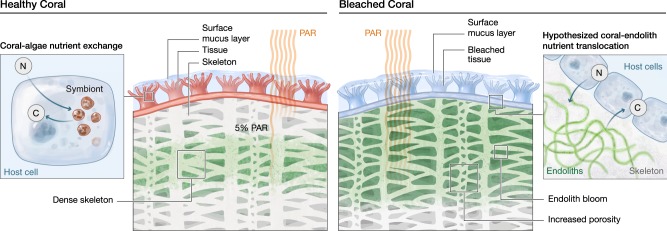
Climate change affects endolithic microbiomes and their interactions with the coral host. **a** In the intact symbiosis, the nutrient exchange between coral cells and the endosymbiotic microalgae is maintained. In this scenario, endoliths remain deep in the skeleton. **b** Exposure to prolonged high-temperature anomalies causes the loss of Symbiodiniaceae from coral tissues, resulting in bleaching. Subsequently more light penetrates into the skeleton, causing endolithic microbiomes to bloom. Endoliths were previously shown to increase their biomass and primary production, physically reaching the animal tissues, and enhance the rates of organic carbon (photoassimilate) translocation to the animal host. It is hypothesized that nutrient exchange between the coral host and endoliths may potentially help the coral animal to survive or even to recover from bleaching. At the same time, the increased growth of endoliths may cause microbioerosion to intensify, undermining the structural integrity of the coral skeleton, and rendering the coral colony more vulnerable to breakage (e.g., during storm events)

The ecological consequences of endolithic blooms in the coral skeleton following bleaching, however, may be complex, and not necessarily exclusively beneficial (Fig. 2). Rather, the higher abundance of endoliths may result in stimulated bioerosion rates, increasing the porosity of the coral skeleton [91, 92]. Indeed, increased abundances and microbioerosion by *Ostreobium* spp. were previously reported under elevated *p*CO2 and ocean warming simulations [91, 93, 94]. Thereby, while endolithic communities may sustain the coral host with a critical supply of organic carbon during bleaching, they may also slowly weaken the structural integrity of the skeleton, potentially rendering the entire coral colony more vulnerable to mechanical damage. Considering that the effects of ocean warming inevitably include the increasing frequency and severity of storms, endoliths may ultimately contribute to the degradation and loss of three-dimensional structure of coral reefs.

## Beyond corals: endolithic communities in reef ecosystems

On tropical coral reefs, endolithic microbial communities are not restricted to the coral skeleton, but rather, can occupy a large variety of niches such as coral rock, sand, and other calcifying benthic organisms [5, 55, 95]. For example, endoliths commonly occur in association with crustose coralline algae [96], foraminifera [97], calcifying algae (e.g. *Halimeda* spp.) [97], and mollusc shells [5, 95]. Similar to the communities present in corals, the endoliths reported in these benthic organisms include cyanobacteria, filamentous algae, fungi, and bacteria [5, 95], but a full assessment of their diversity and exact taxonomic composition is currently lacking.

Coral rock (the dead carbonate substrate creating the reef framework) and coral ‘rubbles’ (generated by the progressive breakdown of coral rock into increasingly smaller pieces) are typically colonised by diverse communities of phototrophic eukaryotes, including *Ostreobium* [98]. In addition, the cyanobacteria *Acaryochloris* spp. can thrive in coral rock overgrown by the crustose coralline algae [99]. *Acaryochloris* are known to be the only oxygenic photoautotroph that uses the red-shifted chlorophyll d as their main photosynthetic pigment, enabling them to take advantage of the NIR light conditions [100]. Hence, this genus might be a significant contributor to oxygenic photosynthesis given that its endolithic habitat is particularly common and widespread in coral reefs [99]. Endolithic microorganisms are therefore considered the main driver of photosynthesis in abundant dead carbonate substrates [55], but the impact of their primary production at the ecosystem scale remains unknown.

In addition, sediments also constitute important microbial habitats and can represent a substantial fraction of reef substrates. The microbial environment of reef sediments is influenced by water motion, grazing pressure, and benthic productivity [101], which create fluxes in oxygen, nutrients, and DOC, potentially affecting endolithic communities. In addition, abiotic factors such as sediment grain size, surface structure and area, permeability and transparency to light can also affect microbial communities and their activity in reef sands [102]. For instance, carbonate sands were recently shown to exhibit higher nitrogen fixation rates than silicate sands in coral reefs of the Gulf of Aqaba, Jordan [102]. Notably, nitrogen fixation rates were positively correlated with gross photosynthetic rates in carbonate sands, but these variables were negatively correlated in silica sand, likely because of differences in their diazotroph communities [102]. In addition, eukaryotic microalgae such as diatoms [103] and dinoflagellates [104], including Symbiodiniaceae, are abundant in reef sediments [104–106]. While it remains unclear whether Symbiodiniaceae simply adhere to sand grains, or exhibit a microboring lifestyle in the reef, they can calcify and populate spherical calcium carbonate structures in association with Gammaproteobacteria in cultures [107], which suggest that Symbiodiniaceae might also inhabit reef sediments as endoliths.

## Future directions

Despite recent progress in our understanding of the taxonomy [19, 50] and structure [21, 108] of endolithic communities, many questions remain yet to be answered. In particular: (a) Does microscale heterogeneity, driven by chemical, light, or pH gradients, spatially stratify the endolithic microbiomes into distinct taxonomic assemblages? (b) What are the functional roles of endolithic communities and do their functional capacities vary spatially in situ? (c) If communities are spatially structured, do the metabolic processes occurring in specific skeleton sections influence neighbouring communities and by extension the coral host? (d) Do the endoliths sustain or harm their coral host during stressful events? Unfortunately, due to the compact nature of the skeleton, most studies to date have relied on the use of hammer and chisel, which result in sampling efforts with coarse spatial resolution and potential loss of microscale heterogeneity by accidental homogenisation. The highest sampling resolution to date followed a geometric progression design on massive *Porites* spp. colonies (with distance ranging from 0.4 to 199.2 cm) and observed high level of microbial taxonomic heterogeneity [21]. Similar fine-scale sampling approaches should, therefore, be emulated and could even be improved in future studies, as outlined below.

**Fig. 3:**
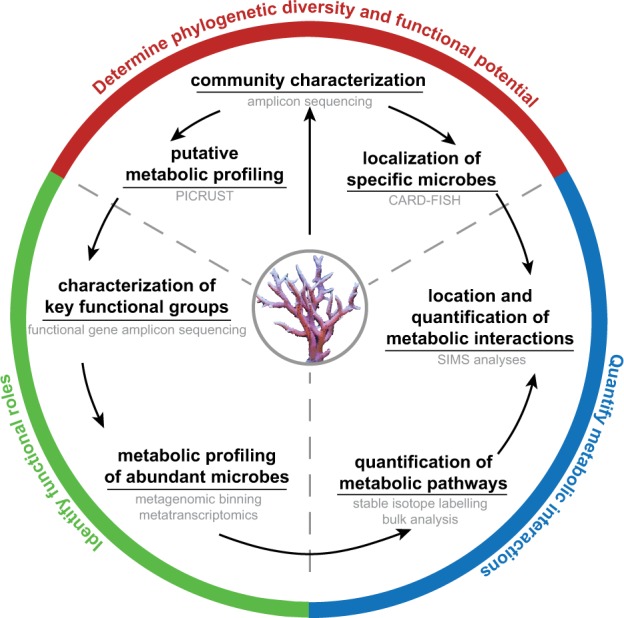
Proposed workflow to untangle the metabolic interactions occurring between microbes in the coral skeleton and between skeleton and tissue communities. Methods used at each step are outlined in grey

Ideally, (1) a comprehensive characterization of skeletal microenvironments and associated gradients (e.g., light levels, nutrient concentrations, pH) using micro-sensors and hyperspectral imaging should clearly identify species-specific “skeletal micro-regions”, i.e. distinct regions or bands prior to sampling. This characterisation could be done by physically taking representative colonies into the laboratory and making large cross sections, using a diamond blade, which could then be measured. (2) After collecting a skeletal cross-section, researchers should subsample it using “hollow punching” of a suitable diameter that would target the predefined “skeletal micro-regions”. This approach will enable the determination of phylogenetic diversity and functional potential of these spatially coherent regions using a combination of marker-based high-throughput amplicon sequencing and –omics approaches (e.g., metagenomics, transcriptomics). (3) Finally, confirming community spatial structures and metabolic exchange would be paramount. This could be achieved via a novel sample preparation technique suitable for high-resolution imaging that retains the structural integrity of the skeleton, coral tissues, and all associated microbes. Most imaging techniques require relatively thin and flat sections, which were until recently impossible to achieve with hard and brittle calcium carbonate skeletons. However, a recent methodological breakthrough now enables unconstrained access to the spatial structure of cryptic endolithic communities by directly cutting micrometre-thin sections of frozen coral samples without the need for prior decalcification [109]. Leveraging on such new methods, researchers could conduct a targeted localization of specific microbial taxa using in situ hybridization on these thin coral cross-sections, or image and quantify the transfer of metabolites using stable isotope probing and NanoSIMS between different skeletal micro-regions and even between the skeleton and the coral tissue. These integrated approaches (Fig. 3) would undoubtedly help to identify the roles endolithic communities play in healthy coral holobionts, as well as during stressful events such as coral bleaching.
